# Is sleep apnea underdiagnosed in adult patients with osteogenesis imperfecta? –a single-center cross-sectional study

**DOI:** 10.1186/s13023-018-0971-7

**Published:** 2018-12-29

**Authors:** Heidi Arponen, Adel Bachour, Leif Bäck, Helena Valta, Antti Mäkitie, Janna Waltimo-Sirén, Outi Mäkitie

**Affiliations:** 1 Department of Oral and Maxillofacial Diseases, University of Helsinki, P.O. Box 41, FI-00014 Helsinki, Finland; 2 Sleep Unit, Heart and Lung Center, Helsinki University Hospital and University of Helsinki, Helsinki, Finland; 3 Department of Otorhinolaryngology – Head and Neck Surgery, Helsinki University Hospital and University of Helsinki, Helsinki, Finland; 4 Children’s Hospital, University of Helsinki and Helsinki University Hospital, Helsinki, Finland; 5 Folkhälsan Institute of Genetics, University of Helsinki, Helsinki, Finland; 6 Center for Molecular Medicine, Karolinska Institutet and Clinical Genetics, Karolinska University Hospital, Stockholm, Sweden

**Keywords:** Osteogenesis imperfecta, Sleep apnea, Osteoporosis, Fatigue, Hypoxia

## Abstract

**Background:**

Patients with Osteogenesis imperfecta (OI) suffer from increased bone fracture tendency generally caused by a mutation in genes coding for type I collagen. OI is also characterized by numerous co-morbidities, and recent data from questionnaire studies suggest that these may include increased risk for sleep apnea, a finding that lacks clinical evidence from cohort studies. In this cross-sectional study, 25 adults with OI underwent clinical otorhinolaryngology examination as well as overnight polysomnography to address the question. The participants were aged between 19 and 77 years, and ten of them had mild clinical OI phenotype, seven had a moderately severe phenotype, and eight had a severe phenotype.

**Results:**

We found obstructive sleep apnea (apnea hypopnea index ≥5/h) in as many as 52% of the OI patients in the cohort. Unexpectedly, however, no correlation was present between sleep apnea and daytime sleepiness, experienced bodily pain, severity of OI, Mallampati score, or neck circumference.

**Conclusions:**

Seeing that the usual predictors showed no association with occurrence of sleep apnea, we conclude that obstructive sleep apnea may easily be left as an undetected disorder in individuals with OI. Recurrent nocturnal hypoxia due to episodes of apneas can even affect bone metabolism, thereby further aggravating bone fragility in patients with OI.

## Background

Osteogenesis imperfecta (OI) comprises a group of genetic disorders characterized by bone fragility. Phenotypic classification of OI into five different types is based on clinical and radiographic findings, and a severity range from mild non-deforming to severe and lethal [1]. Some of the extra-skeletal manifestations of OI are persisting daytime sleepiness and fatigue, the underlying causes of which are still poorly understood [1, 2]. Sleep apnea might be one explanatory factor, and self-reported prevalence of sleep apnea among adult OI populations in US and Finland has been similar, 14–15% [1, 3], being higher than in normal population [4]. Cohort studies supporting this finding or reporting the actual prevalence of sleep apnea in patients with OI have not, however, been published.

Sleep apnea is a condition distinguished by pauses in breathing or instances of shallow or infrequent breathing during sleep, resulting in sleep fragmentation and daytime sleepiness [5]. Sleep apnea is classified as central (CSA), obstructive (OSA), or mixed, and the diagnosis relies on clinical symptoms and measures from polysomnography [6]. Particularly in OI, abnormal connective tissue in the upper airway tract may be a risk factor for sleep apnea. Relative size discrepancy between the tongue and the oral cavity may not only cause bimaxillary labial proclination of incisor teeth and open bite in severe OI types [7], but also narrowing of the oropharyngeal airway and obstruction at sleep. Furthermore, in the severe OI types, trunk deformities and consequent impairment of pulmonary function may contribute to more severe hypoxia related to sleep disordered breathing [1, 8, 9].

Of particular interest with regard to OI are the consequences of eventual sleep apnea to bone. Apneic episodes cause hypoxia, which in turn leads to metabolic changes that are harmful to bones [5]. A number of researchers have reported an association between sleep apnea and low bone mass, but the supporting evidence is subject to debate [10, 11]. Osteoporosis and vertebral fractures, on the other hand, have been linked to increased prevalence of OSA as a secondary consequence [12, 13]. The possible bidirectional causality between disorders of bone metabolism and sleep apnea, however, remains, to the best of our knowledge, unexplored.

In this single-center cross-sectional study, we examined adults with OI clinically and performed an overnight polysomnography to test the hypothesis that undiagnosed central or obstructive sleep apnea is common in this group of patients. Such finding would be significant in two ways: Firstly by giving one explanatory reason for the excessive fatigue reported by adults with OI, and secondly by the importance of diagnosis and proper treatment of sleep apnea that might aggravate bone fragility in OI.

## Methods

**Fig. 1:**
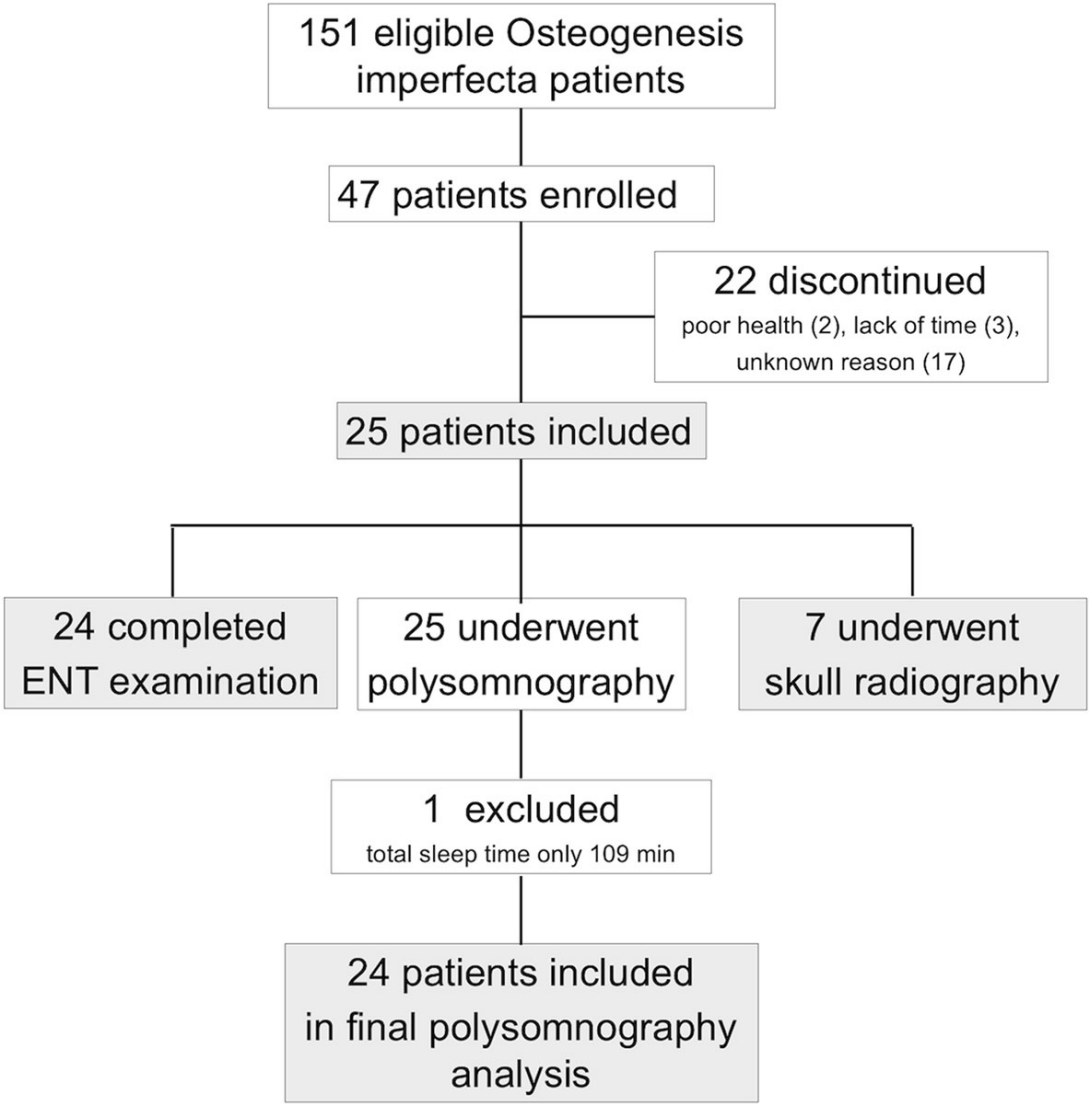
Flow-chart of patient inclusion in the study

The study was approved by the Research Ethic Board of Helsinki University Hospital, Helsinki, Finland (12/18/2014). We sent an invitation letter in January 2015 to all 151 adult patient members of the Finnish Osteogenesis Imperfecta Society to participate in a study entity exploring the quality of sleep and its relation to daytime well-being. Forty-seven individuals initially enrolled. Of them, 25 were willing and able to participate in the present study, comprised of clinical examination and polysomnography, during its course between September 2016 and April 2018. Informed consent was obtained from all study subjects. One participant had undergone a polysomnography as part of normal clinical care and the results of that assessment were included in the study; otherwise the patient participated according to the study protocol. Figure 1 presents a flow chart of the patient inclusion. The participants represented OI types I (*N* = 10), III (*N* = 8), and IV (*N* = 7), as classified according to the original Sillence classification [14]. Information on participant genotype was not available.

Epworth Sleepiness Scale (ESS) was used to evaluate daytime sleepiness, and a validated self-rating depression questionnaire (DEPS) to identify possible depression [15–17]. Visual analogue scale (VAS) from 0 (no pain) to 10 (worst imaginable pain) was used to assess self-reported bodily pain [18].

All patients but one, who chose not to attend the clinic appointment, underwent a clinical ear, nose, and throat examination, performed by an otorhinolaryngologist – head and neck surgeon to evaluate upper airway structures and patency. It included evaluation of clinical morphology of the oropharynx, as described by Bäck et al. [19]. Patients were measured for standing height, weight, and neck circumference; body mass index (BMI) was calculated based on height and weight and expressed as kg/m^2^. For the ten wheelchair-bound patients, self-reported height was recorded. Lateral cephalometric radiographs in a standing position were taken from seven patients, while the others declined from radiographic examinations. The radiographs were analyzed for craniofacial morphology and the results were compared with age- and gender-matched Bolton standards [20].

A full overnight polysomnography in sleep laboratory was performed using Embla N7000 (Natus, Middleton, WI USA), including 6-electrode encephalography, electrooculography, electromyography, oximetry, thermistor, pressure cannula measurements of airflow, and measurements of ribcage and abdominal movements by plethysmography. Transcutaneous CO_2_ (TcCO_2_) was measured with SenTec (SenTec Digital Monitoring System, Therwil, Switzerland). We applied the scoring guidelines and sleep apnea definition of the American Academy of Sleep Medicine [21, 22]. Apnea was defined as complete cessation of airflow for at least 10 s. Hypopnea was defined as a reduction of ≥30% in the airflow signal for at least 10 s associated with oxygen desaturation of ≥3% or arousal. Apnea-hypopnea index (AHI), signifying the number of apneic and hypopneic events per hour of sleep, was calculated to indicate the severity of sleep apnea. According to American Academy of Sleep Medicine Standards, no OSA is present when AHI was less than 5 per hour, a mild OSA when AHI is ≥5, but < 15 per hour, a moderate OSA when AHI ≥ 15, but < 30 per hour, and a severe OSA when the polysomnography demonstrates AHI ≥ 30 per hour [23]. The monitored values during wake-state and sleep were compared with published normal values [23]. The patients’ statural heights were converted to age- and sex-specific Z-scores according to Finnish references [24], and the Z-scores were used in the statistical analysis as an expression of clinical severity of OI. Continuous variables are reported as median (range). Spearman’s rank correlation and linear regression analysis was calculated to assess the association between the variables. Difference between group means was examined with one-way ANOVA analysis.

## Results

Of the 25 participants, 8 were males and 17 females. The median age was 48 years (range 19 to 77 years). All participants were ethnic Finns and represented OI of various severity (Table 1). Ten patients used wheelchair as primary means of mobility aid and 15 were independent walkers. The height Z-scores median was − 2.2 (range 1.7 to − 12.5). Seventeen of the patients exceeded the WHO criterion for adult overweight of BMI ≥ 25. However, only 12 of the 25 participants had normal height, and of them six were overweight. Neck circumference was measured in 11 patients and ranged from 23 to 51 cm, with a median of 35.5 cm. Lower anterior facial height, which is the distance between the most anterior point on maxillary bone and lowest point on mandibular symphysis, was measurable on five out of the seven lateral skull radiographs. In the other two, mandibular symphysis fell partly below the field-of-view because of difficult positioning of the short-necked patients within the cephalostat. The value was below the norm in four patients and exceeded the norm in one patient by 2 mm. The craniofacial growth pattern was vertical in three out of the seven patients with lateral skull radiograph obtainable.

**Table 1:** Osteogenesis imperfecta (OI) patient characteristics, results of questionnaires, clinical ear, nose, and throat examination, and cephalometric analysis

Patient	Age	OI type	Gender	Height z-score	BMI	ESS	DEPS	Pain	Neck circumference	Mallampati score	Uvula grade	Tonsil size	Pharyngeal webbing	Tongue base	LAFH	SN/MP
			M/F		kg/m2				(cm)						mm	degree
1	27	III	F	−9.5	37.6	11	NA	1	29	1	1	3	2	3		23
2	31	III	F	−5.0	25.1	4	NA	7	45	1	1	2	2	3	54	34
3	39	I	F	−2.7	31.2	18	NA	5	36	3	1	0	1	2		
4	52	III	M	−9.0	43	11	6	7	51	1	3	2	3	3	54	28
5	49	I	M	−1.1	33.6	13	14	7	48	2	1	1	2	1		
6	72	I	F	−1.2	21.1	6	NA	2	39	2	1	1	1			
7	47	I	F	−0.3	22.1	13	NA	8	23	1	1	1	2	1	69	40
8	55	IV	F	−1.7	26.8	8	2	1	33	2	1	1	1	1		
9	54	IV	M	−1.7	26.9	5	18	4	NA	3	2	2	2	1		
10	28	III	F	−5.2	40.1	5	2	2	NA	1	3	3	2	1	62	30
11	44	I	F	−2.3	26.7	11	1	7	NA	2	1	2	1	1		
12	49	IV	F	−10.7	29	4	1	7	33	3	2	2	2	1		
13	19	III	M	−8.0	20.2	10	3	3	32	4	2	2	1	2	52	47
14	30	I	F	−1.8	32	13	17	5	NA	3	1	1	1	1		
15	77	I	M	−2.1	24.9	6	9	4	NA	3	1	1	1	1		
16	46	III	M	−6.6	33.4	4	7	4	NA	3	3	1	1	1		
17	52	IV	F	0.1	22.4	11	6	2	NA	3	1	1	1	1		
18	48	I	F	1.7	20.8	8	5	3	NA	3	1	1	3	0		
19	63	IV	M	−1	26.7	3	4	5	NA	1	1	1	2	1		
20	71	III	M	−3.3	32.1	14	11	9	NA	4	3	2	2	1		
21	31	I	F	−0.7	27.3	11	11	2	NA	3	1	1	2	1		
22	58	IV	F	−2.3	26.4	1	13	5	NA	3	1	2	2	1		
23	36	IV	F	−0.3	21.7	1	3	0	NA	3	2	2	2	1		
24	29	I	F	−1.0	23.4	9	5	NA	NA	NA	NA	NA	NA	NA		
25	40	III	F	−12.5	29.5	5	21	5	50	1	1	1	NA	NA		40
Average	46			−4.0	28	8	8	4	37	2	2	2	2	1		
SD				3.8	6.1	4	6	2	8.8							

Abbreviations: OI type I: Mild, non-deforming; OI type III; Moderate to severe, progressively deforming; OI type IV; Moderate with wide variety in severity; NA classification not performed;

Height Z-score: relative height as SD from gender-specific population median; BMI: body mass index;

*ESS* Epworth sleepiness scale; *DEPS* Depression Scale (0–30 score scale); Pain: as evaluated by VAS scale;

Neck circumference: at the level of the cricothyroid cartilage; Mallampati score: soft palate position, scale 1–4; Uvula grade: the size of uvula, scale 1–4; Tonsil size: grade 0, the tonsils removed, size scale 1–4; Pharyngeal webbing: scale 1–4; Tongue base: 1 open, 2 tip of epiglottis visible but not vallecula, 3 vallecula not visible at all

*LAFH* lower anterior facial height

*SN/MP* Angle between sella-nasion plane and mandibular plane

In the clinical examination, Mallampati score was 3 or 4 in 13 patients, six of whom turned out to suffer from sleep apnea (Tables 1 and 3). None of the patients exhibited large size grade 4 uvula, grade 4 pharyngeal webbing, or tonsils extending to the midline. None of the patients had nasal obstruction affecting both nasal passages. In three patients, the tongue base was hyperplastic and/or the bottom of the vallecula was not visualized, raising the clinical suspicion of obstruction at the tongue-base level. Two of them were later confirmed to suffer from sleep apnea.

An abnormally high ESS value of 10 or above, suggesting increased daytime sleepiness, was found in 11 of the patients (Tables 2 and 3). In five patients, DEPS scale showed a result higher than 12, previously indicated as the cut-off point for clinical depression. Average VAS score for pain was 4 (range 0 to 9). No correlation was found between bodily pain, daytime sleepiness, or depression score. Three of the patients used occasionally insomnia medication.

**Table 2:** In-laboratory polysomnography findings

Patient	TST	SE	Arousals	AHI	AHI	AHI	SpO_2_	CT90	ODI3	PLMI	Snoring time
	minutes	%	total	total /hour	obstructive /hour	central /hour	sleep mean %	%	/ hour	/ hour	% of TST
1	383	94	21.9	21.4	21.4	0.0	92.6	7.1	24.9	6.8	34
2	252	63	13	3.6	3.6	0.0	95.4	0.0	1.9	0.0	22.7
3	274	65	6.2	0.2	0.2	0.0	97.1	0.0	0.0	3.3	18.1
4	294	73	55	45.1	45.1	0.0	91.5	17.3	42.0	0.0	51.5
5	282	76	30.3	29.5	28.0	1.5	93.0	1.6	16.9	1.7	67.7
6	304	78	24.9	23.1	22.7	0.4	92.5	2.4	12.0	0.0	26.5
7	371	79	8.6	0.0	0.0	0.0	95.4	0.0	0.0	15.9	3.6
8	295	74	8	0.6	0.6	0.0	95.0	0.0	3.3	8.4	5.7
9	109	32	81.3	80.2	80.2	0.0	92.6	0.4	25.2	11.2	66.2
10	424	87	4.8	0.3	0.3	0.0	94.6	0.0	0.3	3.8	0.3
11	409	95	24.1	1.9	1.8	0.1	96.2	0.0	0.6	0.0	0.9
12	256	57	47.4	18.5	18.5	0.0	92.5	3.7	18.0	8.0	3.7
13	371	93	15	0.2	0.2	0.0	92.6	0.0	0.2	2.3	0
14	315	68	12.4	1.0	1.0	0.0	93.7	0.0	0.2	0.0	3.6
15	310	78	19.6	14.2	14.2	0.0	93.2	0.8	11.8	0.0	33.6
16	299	81	20.5	7.5	7.5	0.0	94.8	0.1	2	2.2	48.3
17	435	75	11.6	5.8	5.6	0.2	94.9	0.0	4.3	0	29.4
18	362	88	37.2	3.8	3.8	0.0	95.7	0.0	0.8	10.6	0.8
19	327	78	23.5	10.7	10.7	0.0	93.8	0.3	7.0	68.4	3.4
20	320	72	18.8	24.8	24.8	0.0	93.3	4.4	27.8	53.4	45.8
21	306	71	25.5	1	1	0.0	96.8	0.0	2.7	7.8	0
22	379	80		24.9	24.9	0.0	94.6	0.6	19.2	43.0	26.6
23	399	94	13.8	0.6	0.6	0.0	95.9	0.0	0.2	2.4	0
24	343	79	23.3	11.4	11.4	0.0	96.1	0.1	2.8	0.0	6.1
25	403			10			90.6	23.0			
mean	329	76	24	14.0	14.0	0.1	94.2	2.5	9.3	10.4	20.1
SD	70	14	18	19	19	0.3	1.6	3.8	11.5	18.1	22.0

Abbreviations: *TST* total sleep time; *SE* sleep efficiency; *AHI* apnea and hypopnea index

SpO_2_: pulse oxygen saturation in blood; CT90: SpO_2_ cumulative time below 90%; PLMI: periodic leg movement index

Patient number 9 was left out of the further analysis due to insufficient sleep efficiency

**Table 3:** Summary of the results by Osteogenesis imperfecta (OI) type

	OI type			
	Mild (type I)	Moderate (type IV)	Severe (type III)	Total number of patients
N	10	7	8	25 (100%)
Sleep apnea confirmed (AHI > 5/h)	4	4	5	13 (52%)
Overweight (BMI ≥ 25)	5	5	7	17 (68%)
Abnormally high ESS (≥10)	6	1	4	11 (44%)
Mallampati score 3 or 4	5	5	3	13 (52%)

Abbreviations: *AHI* apnea and hypopnea index; *BMI* body mass index; *ESS* Epworth sleepiness scale

**Fig. 2:**
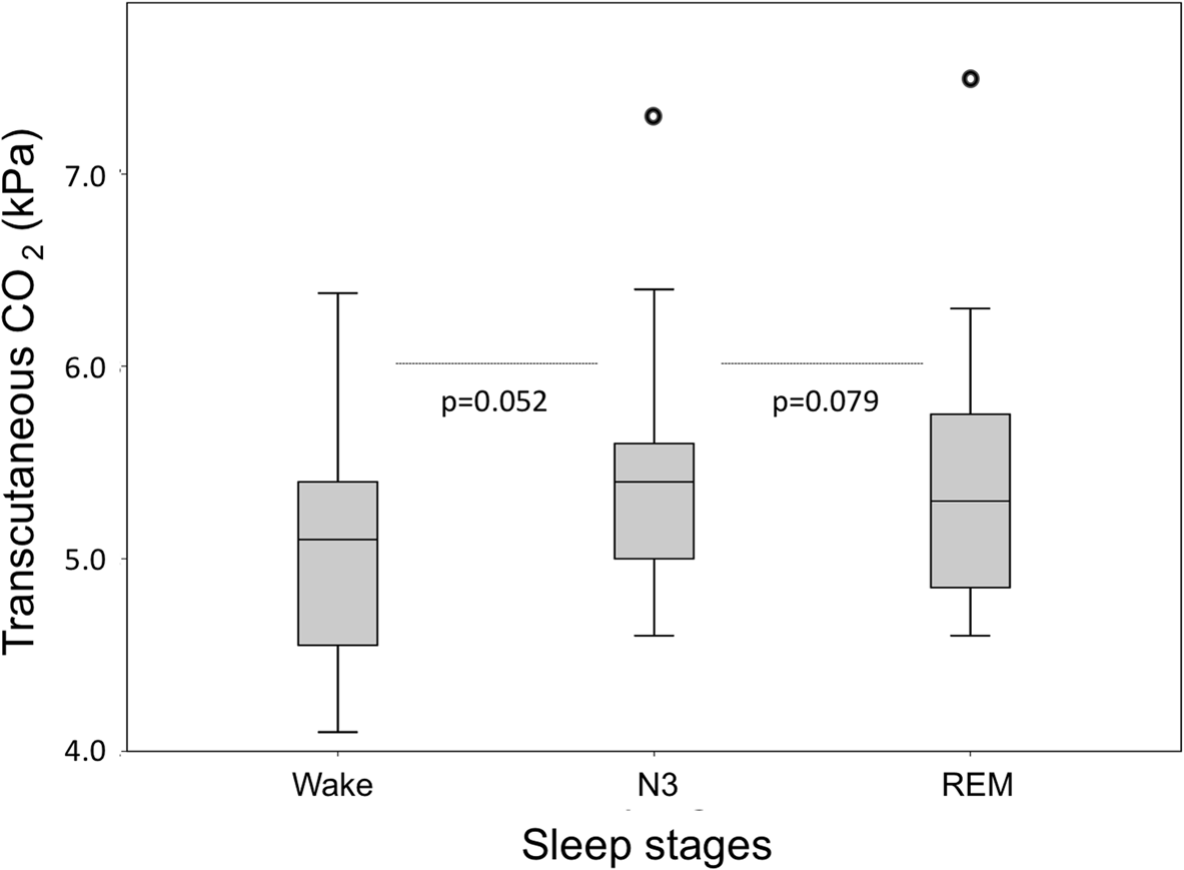
Mean transcutaneous CO_2_ (TcCO_2_) values in patients with Osteogenesis imperfecta during the in-laboratory polysomnography study. TcCO_2_ was recorded on 23 patients during wake-state, N3 sleep-stage, and REM sleep-stage. There was no significant increase in the TcCO2 values during sleep compared to wakefulness (*p* > 0.05)

**Fig. 3:**
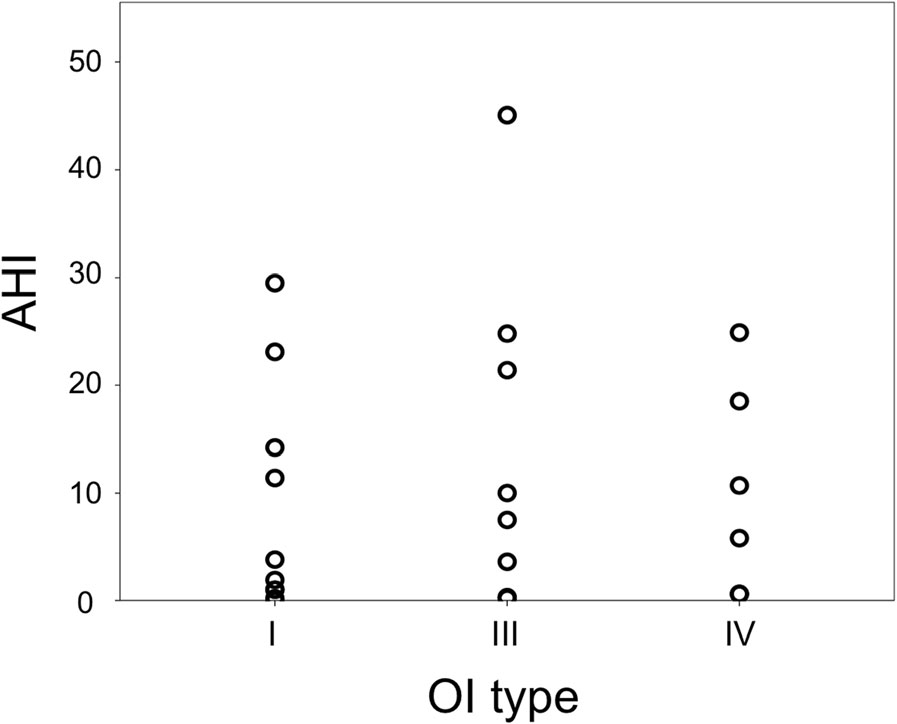
Apnea and hypopnea index (AHI) according to Osteogenesis imperfecta (OI) type. The scatter plot illustrates the relationship between AHI and OI type for 24 study participants. AHI is the number of apneas or hypopneas recorded during the study per hour of sleep. Each circle represents the measured value for an individual patient. No correlation was found between AHI and OI type (*p* = 0.618)

One patient was excluded from the analysis due to the total sleep time being less than 120 min (patient number 9 on Table 2). The patient in question had been diagnosed with sleep apnea years earlier but no treatment had been undertaken. TcCO_2_ recording was unsuccessful for one patient (patient number 25 on Table 2). Table 2 and Figs. 2 and 3 present the successful recordings of the polysomnography. In 13 of the patients (52%) the sleep analysis was diagnostic for OSA. Six of them had mild OSA, six had moderate OSA and one a severe OSA. ODI, i.e. hourly average number of desaturation episodes, ranged from 2 to 42 in those patients who got a sleep apnea diagnosis. Respiratory events were almost exclusively of obstruction origin. No correlation was found between AHI and pain, daytime sleepiness, severity of OI (Fig. 3), Mallampati score, neck circumference, BMI, or height Z-score. Those with severe OI had higher size grading of tonsils, uvula, and tongue base indicating more obstructed airways (*p* < 0.05). When an increase in AHI was predicted, we found that high BMI was a significant predictor (Beta = 0.89, *p* < 0.05). The other variables were not significant predictors (*p* > 0.05). However, the overall linear regression model fit was weak (R-squared 0.2). The difference between the wake-state and N3 sleep-stage TcCO_2_ levels was not statistically significant (mean 5.04 vs. 5.40 kPa respectively) [F(1, 45) = 4.007, (*p* = 0.052)] (Fig. 2). Likewise, the difference between N3 sleep-stage and REM sleep-stage TcCO_2_ was not significant (mean 5.40 vs. 5.42 kPa respectively) [F(1, 45) = 0.012, (*p* = 0.0914)].

## Discussion

To our knowledge this is the first systematic study on sleep apnea in an unselected adult OI population. Half of the participants with OI showed with an abnormal apnea and hypopnea index during an overnight polysomnography, indicating a high frequency of sleep apnea in this patient population. Despite the apneic and hypopneic events, the mean sleep-time oxygen saturation levels stayed, however, at 93% in those with sleep apnea, which is an acceptable level. Similarly, the patients also displayed normal transcutaneous CO_2_ levels both during wakefulness and sleep.

Generally recognized factors predisposing to OSA are obesity, snoring, male gender, structural abnormalities in the nose, pharynx, and jaws, as well as functional factors such as decreased tidal volume and disturbances of ventilation [25]. Obesity as such is associated with objective and subjective daytime sleepiness, compared to normal-weight controls, regardless of sleep apnea and sleep loss [26]. Many of our patients displayed high BMI, but the use of BMI can be misleading in individuals with short stature, scoliosis and lower extremity deformities. Previous studies have indicated that location of fat deposition, especially anterolateral to the upper airway, is more important than BMI regarding the risk to OSA [27]. Fat distribution was not evaluated in this study. Neck circumference has also been suggested to reflect the risk of OSA, the risk nearly doubling by a 2.5 cm increase [28]. In our sample, no correlation between neck circumference and AHI was detected, suggesting that other factors than fat deposition in the neck region play a role in sleep apnea pathogenesis in our OI-patients. Looking further at the common predictors of OSA, it is notable that of those who acquired the diagnosis in our OI cohort, half were females and sleep apnea was diagnosed from the age 27 years onwards. Most of the patients snored during the polysomnography for a mean period of one fifth of the sleeping time, which indicates some level of upper airway obstruction during sleep.

In patients with OSA, anatomic narrowing may occur at different levels of the upper airway, but the most common locations are in the oropharynx and hypopharynx [25]. The Mallampati method evaluates the relationship between the position of the tongue and the oral cavity. Its clinical usefulness has been proven as an independent predictive factor of obstructive sleep apnea, alongside tonsil size grading, neck circumference, and BMI [29]. The mean Mallampati score was relatively low, in our cohort, although the frequency of sleep apnea was high. Interestingly, in our cohort, only one of the two patients with the highest Mallampati score 4, indicating a possible obstruction site at the level of tongue base, suffered from sleep apnea. In six out of the 11 patients with an abnormal ESS values sleep apnea was not confirmed by the sleep study, suggesting other causes of daytime sleepiness than sleep apnea.

An increase in lower anterior facial height and a vertical growth pattern have previously been identified as craniofacial features contributing to susceptibility to OSA in adults [30]. The paucity of cephalometric material makes analysis difficult, but if anything, it shows tendency to the opposite craniofacial pattern. In general, applicability of cephalometric analysis in evaluating a risk of OSA in patients with OI is likely to be poor due to the specific craniofacial characteristics associated with particularly the severe disorder types [7].

In patients with OI, laryngomalacia has been shown to be a cause of sleep apnea [31]. None of our patients had laryngomalacia. Patients with another heritable connective tissue disorder, Marfan syndrome, have more often sleep apnea than controls, likely due to the laxity of connective tissue blocking the airways in supine position [32]. In severe types of OI, the patients manifest scoliosis and vertebral deformities that decrease body height and impede lung function. Restricted lung function has been linked to the severe OI forms and to increased mortality risk due to respiratory diseases [6, 33, 34]. In our patients, no hypoxia or hypercapnia was found while awake, excluding severe respiratory insufficiency in the cohort.

These results need to be interpreted with caution, bearing in mind a possible sample bias, since individuals that suffer from daytime sleepiness or sleep disturbances would be more likely expected to enroll in the study leading to a prevalence overestimation. A limitation of our study is the small cohort size due to rarity of OI. The paucity of data on neck circumference and skeletal jaw relationships weakened their statistical analysis.

## Conclusions

Daytime sleepiness is a commonly experienced problem by individuals with OI affecting their daily life. Sleep apnea, on the other hand, is a major public health problem, and a significant cause of persisting sleepiness. In contrast to earlier studies, based on self-reported inventories, our systematic evaluation found higher frequency of sleep apnea in adult OI population [1]. Neither the clinical findings, nor the patients’ complaints correlated with the presence of sleep apnea, which consequently can result in OSA being left as an undetected disorder in individuals with OI. In these patients, intermittent nocturnal hypoxia, sleep restriction due to episodes of apneas, and increased sympathetic tone related to sleep fragmentation could affect bone metabolism and architecture, possibly further aggravating osteoporosis and bone fragility. Further prospective research on larger patient populations are needed to verify the finding of high prevalence of sleep apnea in individuals with OI, and to further explore its underlying causes and risk factors, as well as to establish clinical guidelines for sleep apnea screening within this special group of patients. Future studies, taking into account chest wall deformities, scoliosis, kyphosis, history of arthrodesis, and skull base abnormalities, would be useful in evaluating lung volume modification favoring OSA in this patient group. The relevance of polysomnography is clearly supported by the current findings.

## Abbreviations

AHI: Apnea-hypopnea index
BMI: Body mass index
DEPS: Self-rating depression questionnaire
ESS: Epworth sleepiness scale
OI: Osteogenesis imperfecta
OSA: Obstructive sleep apnea
TcCO_2_: Transcutaneous carbon dioxide
VAS: Visual analogue scale
